# Re-Evaluation of the Action Potential Upstroke Velocity as a Measure of the Na^+^ Current in Cardiac Myocytes at Physiological Conditions

**DOI:** 10.1371/journal.pone.0015772

**Published:** 2010-12-31

**Authors:** Géza Berecki, Ronald Wilders, Berend de Jonge, Antoni C. G. van Ginneken, Arie O. Verkerk

**Affiliations:** Department of Anatomy, Embryology, and Physiology, Heart Failure Research Center, Academic Medical Center, University of Amsterdam, Amsterdam, The Netherlands; Brigham & Women's Hospital, United States of America

## Abstract

**Background:**

The SCN5A encoded sodium current (I_Na_) generates the action potential (AP) upstroke and is a major determinant of AP characteristics and AP propagation in cardiac myocytes. Unfortunately, in cardiac myocytes, investigation of kinetic properties of I_Na_ with near-physiological ion concentrations and temperature is technically challenging due to the large amplitude and rapidly activating nature of I_Na_, which may seriously hamper the quality of voltage control over the membrane. We hypothesized that the alternating voltage clamp-current clamp (VC/CC) technique might provide an alternative to traditional voltage clamp (VC) technique for the determination of I_Na_ properties under physiological conditions.

**Principal Findings:**

We studied I_Na_ under close-to-physiological conditions by VC technique in SCN5A cDNA-transfected HEK cells or by alternating VC/CC technique in both SCN5A cDNA-transfected HEK cells and rabbit left ventricular myocytes. In these experiments, peak I_Na_ during a depolarizing VC step or maximal upstroke velocity, dV/dt_max_, during VC/CC served as an indicator of available I_Na_. In HEK cells, biophysical properties of I_Na_, including current density, voltage dependent (in)activation, development of inactivation, and recovery from inactivation, were highly similar in VC and VC/CC experiments. As an application of the VC/CC technique we studied I_Na_ in left ventricular myocytes isolated from control or failing rabbit hearts.

**Conclusions:**

Our results demonstrate that the alternating VC/CC technique is a valuable experimental tool for I_Na_ measurements under close-to-physiological conditions in cardiac myocytes.

## Introduction

Voltage-gated Na^+^ channels are responsible for the rapid action potential upstroke of cardiac myocytes and play a vital role in the proper conduction of the cardiac electrical impulse. Consequently, decreased Na^+^ channel function during common pathological conditions [1]–[3], or as a result of administration of antiepileptic and local anesthetic drugs [4], [5] may cause conduction disturbances and potentially life-threatening arrhythmias. In addition, loss-of-function mutations in the SCN5A gene encoding the pore-forming α-subunit of the cardiac sodium channel (Na_v_1.5) have been shown to underlie multiple inherited arrhythmia syndromes, including Brugada syndrome, cardiac conduction disease, sinus node dysfunction, sudden infant death syndrome, and atrial standstill, while SCN5A mutations resulting in a persistent or “late” sodium current have been associated with long-QT syndrome type 3 (for review, see Refs. [6], [7]). Cardiac sodium channel function is modulated by a large number of interacting and regulatory proteins, including the auxiliary β-subunits (β1–4), ankyrins, fibroblast growth factor homologous factor 1B, calmodulin, caveolin-3, 14-3-3η, Nedd4-like ubiquitin-protein ligases, and syntrophins (for review, see Ref. [8]). Consequently, mutations leading to malfunctions of such proteins contribute to various cardiac syndromes [6], [8].

To understand the functional consequences of Na^+^ channel mutations or the effects of altered Na^+^ channel modulation under various pathophysiological conditions and during administration of various drugs, biophysical properties of the Na^+^ current (I_Na_) are usually studied in the whole-cell configuration of the patch clamp technique [9] in heterologous expression systems or freshly isolated cardiac myocytes. However, investigation of Na^+^ channel kinetics with near-physiological ion concentrations and temperature is technically challenging due to the large amplitude and rapidly activating nature of I_Na_, which may seriously hamper the quality of voltage control over the membrane [10]. In heterologous expression systems, accurate determination of Na^+^ channel properties under close-to-physiological conditions is possible by using low-resistance patch-pipettes, adequate series resistance compensation, and by selecting small cells with low ion channel protein expression levels [11]–[17]. However, in freshly isolated cardiac myocytes, the large I_Na_ is routinely investigated under far-from-physiological conditions (e.g. low temperature and low extracellular Na^+^ concentration) [18], [19] in order to obtain reliable voltage control during recordings. It is obvious that such experimental conditions do not only result in underestimation of the magnitude of the current, but may also alter its biophysical properties [20]. In addition, gating changes of Na^+^ channel mutations may be more prominent at near-physiological temperature than at room temperature [11], [14], [15], [21], [22]–[24]. Furthermore, the effects of antiarrhythmic drugs on Na^+^ channels are markedly altered by temperature [25]. These findings indicate that one should be cautious extrapolating conclusions derived from Na^+^ channel data at low temperatures to clinical situations. Finally, there is evidence that behaviour of Na^+^ channel in cell expression systems may be different from that in myocytes [26], and the increasing amount of mouse models of various cardiac disorders [27] further advertise the need for accurate measurements of I_Na_ and its kinetics under close-to-physiological conditions in cardiac myocytes.

Till now, only few attempts have been made to record cardiac I_Na_ under close-to-physiological conditions. Murray et al. [28] recorded quasi-macroscopic I_Na_ in cell-attached mode using electrodes with relatively large tip openings to record from macropatches on the surface membrane of intact guinea pig ventricular myocytes. They found that temperature clearly affected the voltage dependence of I_Na_ (in)activation, resulting in a more positive membrane potential for the half maximal (in)activation) at higher temperatures [28]. In addition, this macropatch technique was used to characterize the effects of heart failure on brain-type Na^+^ channels in rabbit ventricular myocytes [29]. A disadvantage of the quasi-macroscopic I_Na_ measurements, however, is the inability to measure cellular resting potential at the time the voltage clamp data is collected [28], which is especially important in case of relative large quasi-macroscopic I_Na_ which may depolarize the intact myocytes [30].

In contrast to whole-cell and macropatch I_Na_ measurements in voltage clamp experiments, it is fairly easy to record action potentials under close-to-physiological conditions from cardiac myocytes in the current clamp configuration of the patch clamp technique. The upstroke of the action potential is caused by the flow of ions through channels specific for Na^+^ [31]. Expressed in terms of a simple resistance and capacitance circuit, the change in membrane potential, dV/dt, is proportional to ionic current flow and the steepest portion of the action potential upstroke, dV/dt_max_, occurs at maximal Na^+^ ion flow [32]. Albeit dV/dt_max_ represents the physiological, functional availability of I_Na_, the use of dV/dt_max_ as an adequate measure of Na^+^ channel conductance has been debated, with arguments, either in favour or against, largely based on computer simulation studies (see [33]–[38], and primary references cited therein). It has been concluded by both Hondeghem [35] and Cohen et al. [34] that a future direct comparison between I_Na_ and dV/dt_max_ at physiological temperature and ion concentrations would be very valuable.

In the present study, we evaluated the biophysical properties of I_Na_ expressed in human embryonic kidney (HEK) cells under close-to-physiological conditions with the use of alternating voltage clamp/current clamp (VC/CC) and compared the VC/CC data to data from conventional voltage clamp (VC) experiments in HEK cells, thus validating the use of dV/dt_max_ as an adequate measure of I_Na_. As an application of dV/dt_max_ as a simple tool in studying I_Na_ characteristics under close-to-physiological conditions, we used the VC/CC to characterize I_Na_ in freshly isolated cardiac myocytes of control and heart failure rabbits.

## Methods

### Ethics Statement

This study was carried out in strict accordance with the recommendations in the Guide for the Care and Use of Laboratory Animals of the National Institutes of Health. The protocol was approved by the Committee on the Ethics of Animal Experiments of the Academic Medical Center of the University of Amsterdam (Permit Number: DCA-101772) and all efforts were made to minimize animal suffering.

### Cell preparations

#### HEK cells

Wild-type sodium channel α-subunit construct (0.5 µg) was transfected into QBI-HEK-293A cells (Qbiogene, Heidelberg, Germany) together with β_1_-subunit construct (0.5 µg) using lipofectamine (Gibco BRL, Life Technologies). Transfected HEK cells were cultured in MEM (Earle's salts and L-glutamine) supplemented with nonessential amino acid solution, 10% FBS, 100 IU/mL penicillin, and 100 µg/mL streptomycin in a 5% CO_2_ incubator at 37°C for 2 days. Transfected cells were identified under epifluorescent microscopy using green fluorescence protein (GFP) as a reporter gene. To optimize the conventional voltage clamp measurements, we selected small HEK cells (7.7±0.5 pF (mean±SEM; n = 45)), which tend to exhibit smaller currents and smaller capacitative transients [17].

#### Rabbit ventricular myocytes

Heart failure (HF) was induced in 4-month-old male New-Zealand White rabbits by combined volume and pressure overload in two sequential surgical procedures as described previously [39]. In short, volume overload was produced by rupture of the aortic valve until pulse pressure was increased by about 100%. Three weeks later, pressure overload was created by suprarenal abdominal aorta constriction of approximately 50%. Hearts were excised 3 months after the second operation. At the time of sacrifice, a HF index based on relative heart weight (i.e. heart weight to body weight ratio), relative lung weight (i.e. lung weight to body weight ratio), left ventricular end-diastolic pressure, and ascites was calculated [39]. We performed experiments if at least three of the above parameters were abnormal [40], indicating severe HF. Previous studies from our laboratory demonstrated that HF parameters [39] and important cellular parameters for hypertrophy and ionic remodeling [41] between sham-operated and age-matched non-operated rabbits were similar. Therefore, non-operated age-matched healthy animals served as control. To reduce differences in Na^+^ channel expression [42], midmyocardial cells were isolated by enzymatic dissociation from the apical part of the left ventricular free wall as described previously [43]. Single rod-shaped myocytes exhibiting smooth surfaces and clear cross-striations were selected for measurements.

Small aliquots of cell suspension were put in a recording chamber on the stage of an inverted microscope. Cells were allowed to adhere for 5 min after which superfusion with solution at 36±0.2°C was started. The extracellular solution for HEK cell experiments contained (in mmol/L): NaCl 140, CsCl 10, CaCl_2_ 1.8, MgCl_2_ 1.0, glucose 5.5, HEPES 5.0, pH 7.4 (NaOH). For myocyte measurements, the CsCl of the extracellular solution was replaced by 5.4 mmol/L KCl.

### Electrophysiology

#### Data acquisition

I_Na_ and AP upstrokes were measured in the whole-cell configuration of the patch-clamp technique using an Axopatch 200B amplifier (Molecular Devices Corporation, Sunnyvale, CA, USA) or a custom-made amplifier, capable of fast switching between voltage clamp (VC) and current clamp (CC) modes. Voltage control, data acquisition, and analysis were accomplished using custom software. Signals were low-pass filtered with a cut-off frequency of 5 kHz and digitized at 20 kHz. Series resistance was compensated by ≥80%, and potentials were corrected for the estimated liquid junction potential. The technically challenging I_Na_ measurements at physiological temperature in HEK cells using conventional VC experiments were performed as we described previously in detail [11], [13]. Cell membrane capacitance was determined as described previously [44]. For HEK cell measurements patch pipettes (borosilicate glass; resistance ≈2.0 MΩ) contained (mmol/L): CsCl 10, CsF 110, NaF 10, EGTA 11, CaCl_2_ 1.0, MgCl_2_ 1.0, Na_2_ATP 2.0, HEPES 10, pH 7.2 (CsOH). For cardiac myocyte measurements patch pipettes contained (mmol/L): K-gluc 125, KCl 20, NaCl 5, K_2_-ATP 5, BAPTA 10, HEPES 10; pH 7.2 (KOH).

#### Conventional VC and alternating VC/CC experiments

The current density, voltage dependence of Na^+^ conductance activation, steady-state inactivation, recovery from inactivation, and development of slow inactivation were determined using the voltage protocols depicted in the figures and explained in the Results. The holding potential was −140 mV, except in the protocols for recovery from inactivation and slow inactivation because recovery from inactivation is voltage dependent [19], [33]. In the latter experiments we have chosen a holding potential of −85 mV, a value close to the resting membrane potential of working myocytes.

In alternating VC/CC experiments, cells were clamped with protocols similar to those used in conventional VC experiments. However, after the preconditioning voltage dV/dt was measured by switching to the CC mode of the patch clamp amplifier. Noteworthy, both HEK cells and freshly isolated myocytes display fast depolarizations (in the present study named AP upstrokes) upon switching from VC to CC mode. AP upstrokes were elicited by 1.2× threshold (determined at −140 mV) current pulses through the patch pipette. Maximal upstroke velocity (dV/dt_max_) during VC/CC, offline corrected for the contribution of stimulus current, served as an indicator of available I_Na_.

To determine the activation characteristics of I_Na_, current-voltage curves were corrected for differences in driving force and normalized to maximum peak current. Steady-state activation and inactivation curves were fit using the Boltzmann equation I/I_max_ = A/{1.0+exp[(V_1/2_−V)/k]} to determine V_1/2_ (membrane potential for the half-maximal (in)activation) and the slope factor k. Recovery from inactivation was analyzed by fitting a double-exponential function to the data to obtain the time constants of the fast and the slow components of recovery from inactivation: I/I_max_ = A_f_ ×[1.0−exp(−t/τ_f_)]+A_s_×[1.0−exp(−t/τ_s_)], where t is the recovery time interval, τ_f_ and τ_s_ the time constants of the fast and slow components, and A_f_ and A_s_ the fractions of the fast and slow components, respectively. The time course of current inactivation was fitted by a double-exponential equation: I/I_max_ = A_f_×exp(−t/τ_f_)+A_s_×exp(−t/τ_s_), where A_f_ and A_s_ are the fractions of the fast and slow inactivation components, and τ_f_ and τ_s_ are the time constants of the fast and slow inactivating components, respectively.

#### Action potential clamp experiments

Characteristics of SCN5A currents during an action potential upstroke were tested using the action potential clamp technique. An action potential waveform of a human ventricular cell was simulated using the mathematical model by ten Tusscher, Noble, Noble and Panfilov [45], digitized at 10 kHz, and stored as described previously in detail [44]. The thus obtained action potential waveform was used as command signal under voltage-clamp conditions with a cycle length of 800 ms.

### Statistics

Data are expressed as mean±SEM. Values are considered significantly different if *P*<0.05 in unpaired *t*-test or in Two-Way Repeated Measures of Analysis of Variance (Two-Way Repeated Measures ANOVA) followed by pairwise comparison using the Student-Newman-Keuls test.

## Results

### Biophysical characterization of I_Na_ in HEK cells

To characterize activation, steady-state inactivation, recovery from inactivation and the development of slow inactivation, biophysical properties of I_Na_ were first determined in conventional VC experiments and then in alternating VC/CC experiments, in HEK cells. To evaluate dV/dt_max_ as a tool for studying I_Na_ characteristics under close-to-physiological conditions, results of VC and VC/CC experiments were compared.

#### Current density and voltage-dependence of activation

**Figure 1:**
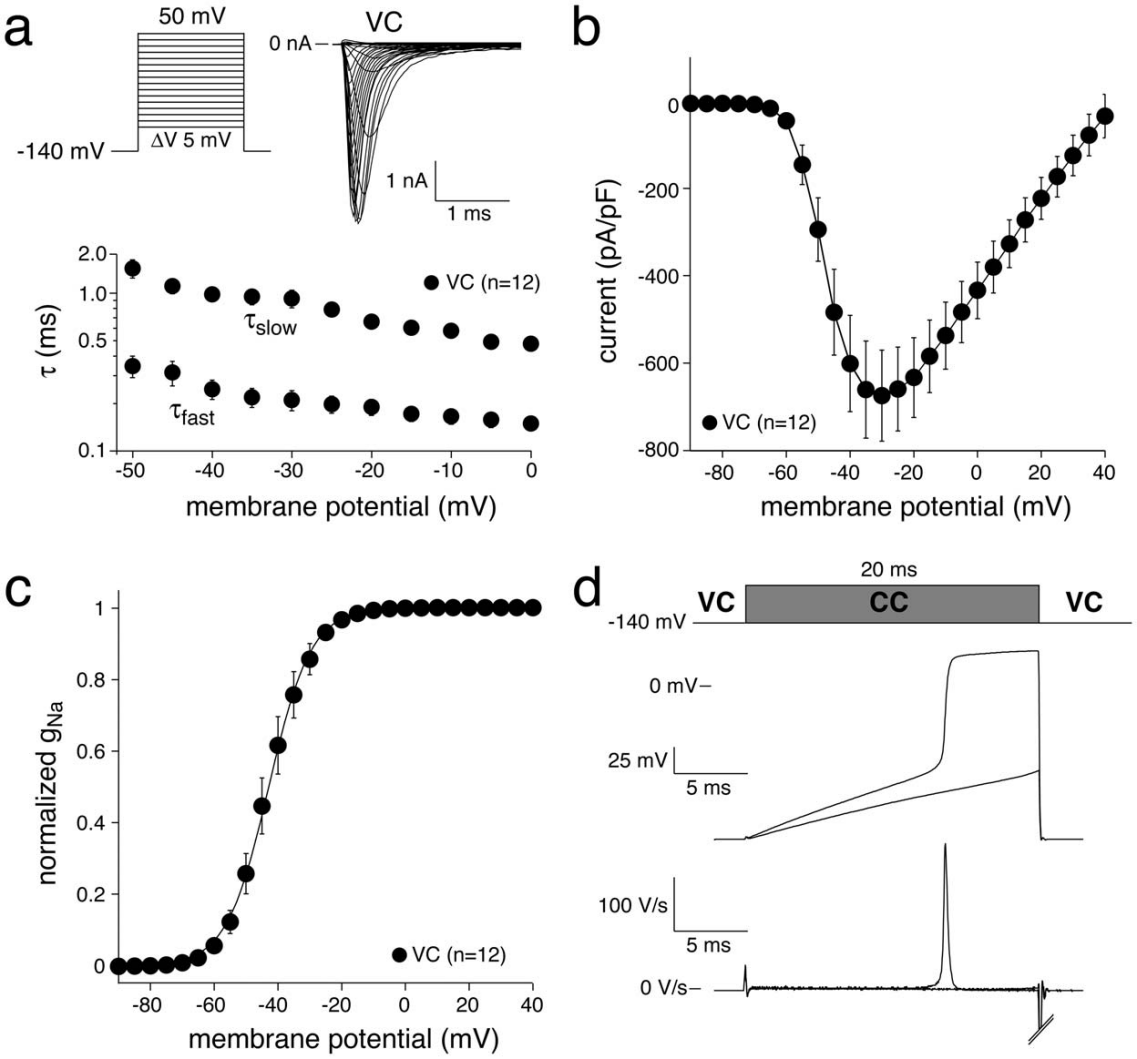
Activation of I_Na_ in HEK cells assessed with square-step voltage protocols in conventional VC experiments. **a**, Top: Typical examples of Na^+^ current in response to depolarizing voltage steps from −140 mV. Bottom: Average fast and slow time constants of Na^+^ current inactivation. Note logarithmic ordinate scale. **b**, Average current-voltage relationship. **c**, Average steady-state activation. The solid line is the Boltzmann fit to the average data. **d**, Typical membrane depolarizations in response to a super- and subthreshold current pulse in a HEK cell during an alternating VC/CC experiment from a holding potential of −140 mV (top) and the first derivatives of the resulting membrane potential changes (dV/dt; bottom).

Figure 1 (top) shows representative I_Na_ activated by 50-ms depolarizing voltage clamp steps of 5 mV increment in a HEK cell using conventional VC. Typical for heterologously expressed SCN5A current measured with square form VC steps, current starts to activate around −60 mV, peaks around −30 mV, and subsequently decreases in amplitude due to the reduction in Na^+^ driving force (Fig. 1b). Figure 1c shows averaged data for the voltage-dependence of ‘steady-state’ activation. Compared to the VC configuration, where I_Na_ characteristics are measured at fixed membrane potentials, in alternating VC/CC experiments the conditioning membrane potential is kept at a fixed (predetermined) value before the recording configuration is switched to the CC recording configuration. Following this switch, activation of I_Na_ will result in an ‘all-or-none’ response, and consequently the cell will depolarize fast to approximately the reversal potential of Na^+^ current [46]. Figure 1d shows the membrane potential in the VC/CC recording configuration in response to a super- and subthreshold current pulse (top) and the first derivative of the membrane potential signal (dV/dt, bottom). Remarkably, the upstrokes in HEK cells are qualitatively similar to AP upstrokes recorded in myocytes.

**Figure 2:**
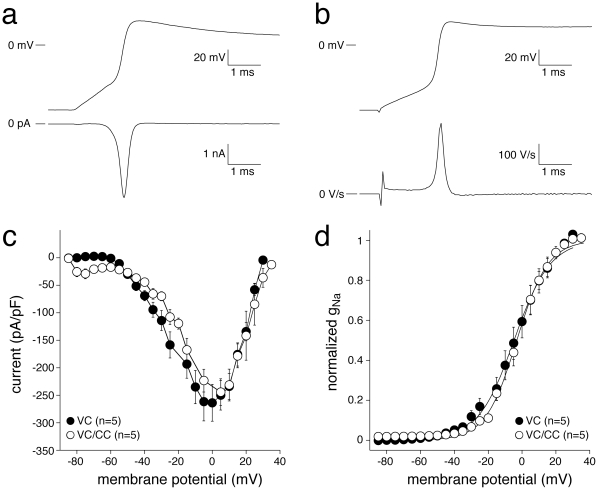
‘Dynamic’ I_Na_ activation in HEK cells assessed with VC (a) and alternating VC/CC (b). **a**, Typical example of Na^+^ current (bottom) during the upstroke phase of the ventricular action potential like waveform used as command potential in an action potential clamp experiment (top). **b**, Typical membrane depolarization from −85 mV (top) in response to a superthreshold current pulse and dV/dt (bottom). **c**, Average phase plane plots with current or dV/dt plotted against membrane potential. Note the similarity between the currrent densities of the VC and alternating VC/CC experiments. **d**, Average dynamic I_Na_ activation. Solid lines represent the Boltzmann fits to the average data.

Although ‘steady-state’ Na^+^ channel activation in alternating VC/CC experiments cannot be determined, AP upstrokes can be used to derive ‘dynamic’ activation properties of I_Na_. To construct dynamic I_Na_ activation properties, phase plane plots (where current density or, equivalently, dV/dt is plotted against membrane potential [47]) are corrected for changes in driving force, current amplitudes normalized to the maximum peak current, and data fitted to a Boltzmann curve to determine membrane potential for half-maximal activation, V_1/2_, and the slope factor, k. In Figure 2 and Table 1, we compare dynamic I_Na_ activation properties measured using AP clamp experiments in the VC mode of the patch clamp amplifier (Fig. 2a) with those of alternating VC/CC experiments (Fig. 2b). Figure 2a shows I_Na_ (bottom) during the upstroke (top) of an action potential clamp experiment. Figure 2b shows dV/dt (bottom) during the upstroke (top) of an alternating VC/CC experiment. Figure 2c shows average phase plane plots. In both VC and alternating VC/CC experiments, I_Na_ activates around −60 mV, peaks around 0 mV and subsequently decreases due to reduced Na^+^ driving force (Fig. 2c). In addition, Figure 2c shows that the current densities did not differ significantly between the VC and alternating VC/CC experiments. The average voltage-dependence of dynamic Na^+^ channel activation in VC and VC/CC experiments is shown in Figure 2d. The dynamic activation properties of I_Na_ measured during VC experiments are almost indistinguishable from those measured during alternating VC/CC experiments, except for a statistically significant difference in the slope factor k (Table 1), indicating that the dynamic I_Na_ activation curve is steeper in VC/CC experiments (cf. Fig. 2d).

**Table 1:** Biophysical properties of I_Na_ at close-to-physiological conditions determined in the VC and VC/CC configurations in HEK cells and in rabbit ventricular myocytes.

	HEK cells		Cardiac myocytes	
	VC	VC/CC	VC	VC/CC
Peak Na^+^ current (pA/pF)	−264±34(n = 5)	−244±24(n = 5)	−438±27(n = 9)	−394±31(n = 8)
Dynamic activation:				
V_1/2_ (mV)	−0.1±3.6	1.4±4.8	5.6±2.8	1.6±2.8
k (mV)	12.4±0.5	9.4±1.0*	13.8±2.8	14.5±2.8
	(n = 5)	(n = 5)	(n = 9)	(n = 8)
Inactivation:				
V_1/2_ (mV)	−84.9±2.2	−84.7±1.4	−74.9±0.8	−74.7±1.5
k (mV)	−5.0±0.1	−5.2±0.5	−4.4±0.2	−4.4±0.2
	(n = 13)	(n = 8)	(n = 17)	(n = 12)
Recovery from inactivation:				
τ_f_ (ms)	6.7±0.7	8.9±1.9	9.6±2.3	9.9±0.8
τ_s_ (ms)	204±51	172±64	131±40	99±27
A_s_/(A_s_+A_f_)	0.24±0.05	0.28±0.05	0.18±0.05	0.21±0.02
	(n = 7)	(n = 7)	(n = 8)	(n = 10)
Slow inactivation				
1-Peak I_Na_ (P2/P1)	0.22±0.03(n = 8)	0.21±0.01(n = 7)	0.11±0.02(n = 14)	0.11±0.02(n = 7)

Mean±SEM, ‘n’ indicates number of cells measured. V_1/2_, membrane potential for half-maximal (in)activation; k, slope factor of steady-state (in)activation curve; τ_f_ and τ_s_, fast and slow time constant of recovery from inactivation, respectively; A_f_ and A_s_, fractions of fast and slow recovery from inactivation, respectively; 1-Peak I_Na_ (P2/P1), fraction of channels that entered into slow inactivation with a P1 of 1 s;

*P<0.05 for CC versus VC/CC in unpaired *t*-test.

#### Voltage-dependence of inactivation

**Figure 3:**
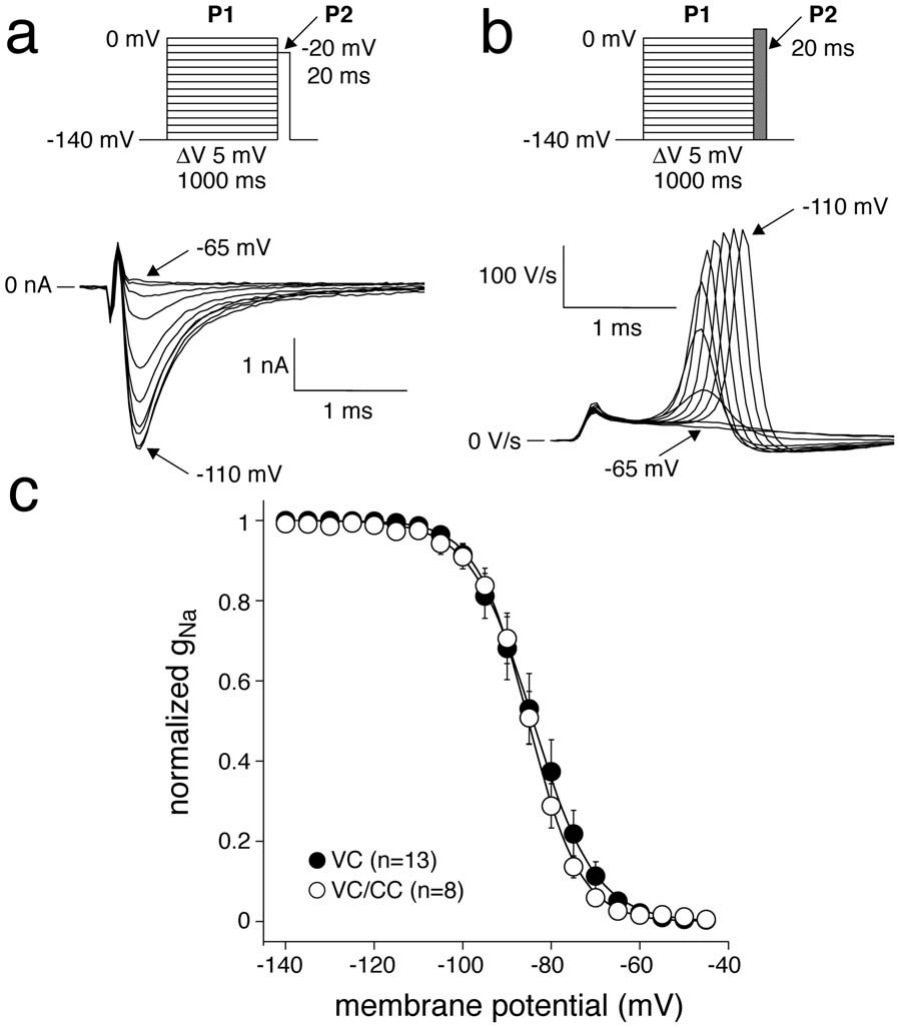
Voltage-dependence of I_Na_ inactivation in HEK cells assessed with VC (a) and alternating VC/CC (b). **a and b**, Top: voltage clamp (a) and VC/CC (b) protocols. Bottom: typical currents (a) and dV/dt's (b) measured after a 1-s prepulse (P1) to membrane potentials between −110 and −65 mV. **c**, Average voltage-dependence of inactivation. Solid lines represent the Boltzmann fits to the average data.

The voltage dependence of inactivation was measured using a two-pulse protocol, where a 1-s conditioning prepulse (P1) to membrane potentials between −140 and 0 mV, to induce steady-state inactivation, was followed by a 20-ms test pulse (P2) (Fig. 3, a and b, top). The bottom panels of Figs. 3a and 3b show typical P2 currents recorded in VC mode and dV/dt values measured in VC/CC mode, respectively. Both peak P2 currents and dV/dt amplitudes were normalized to their maximum values and plotted versus the conditioning pulse (P1) voltage. The thus obtained curves were fitted with a Boltzmann distribution function y = A/[1+exp((V_1/2_−V)/k)] to determine the half-maximal inactivation voltage V_1/2_ (membrane potential at which 50% of Na^+^ channels have entered an inactivated state) and slope factor k of voltage-dependent inactivation. The average data on voltage-dependence of inactivation for VC and VC/CC experiments are shown in Figure 3c and Table 1. The inactivation properties of Na^+^ channels measured during VC experiments were indistinguishable from those measured during VC/CC experiments (Fig. 3c, Table 1).

#### Recovery from inactivation

**Figure 4:**
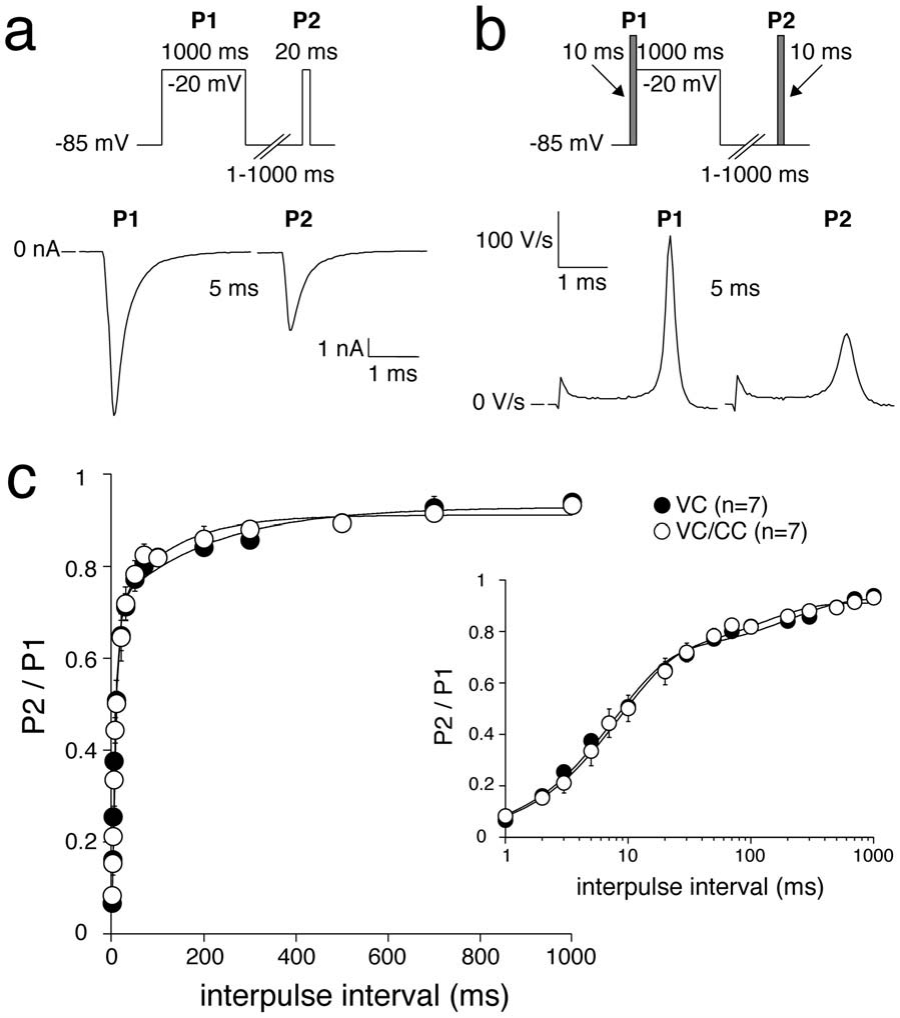
Recovery from I_Na_ inactivation in HEK cells assessed with VC (a) and alternating VC/CC (b). **a and b**, Top: voltage clamp (a) and VC/CC (b) protocols with an interpulse interval of 1–1000 ms, as indicated. Bottom: typical examples of recovery from inactivation with an interpulse interval of 5 ms. **c**, Average recovery from inactivation. Inset, Average recovery from inactivation on a logarithmic time scale. Solid lines are double-exponential fits to the average data.

Recovery from inactivation was measured using a two-pulse protocol, where a 1-s conditioning prepulse (P1) to −20 mV (to inactivate Na^+^ channels) was followed by a test pulse (P2) after a variable recovery interval ranging between 1 and 1000 ms at a recovery potential of −85 mV (Figs. 4a and 4b, top). Figures 4a and 4b (bottom), show typical P1 and P2 currents and dV/dt values, respectively, at an interpulse interval of 5 ms. The peak amplitudes in response to P2 were normalized to the peak amplitudes at P1 and plotted versus the interpulse interval. The resulting curve was fitted with a double-exponential function to obtain the time constants and fractions of the fast and the slow components of recovery from inactivation. Both time constants and fractions of recovery from inactivation were not significantly different in VC and VC/CC experiments (Fig. 4c and Table 1).

#### Slow inactivation

**Figure 5:**
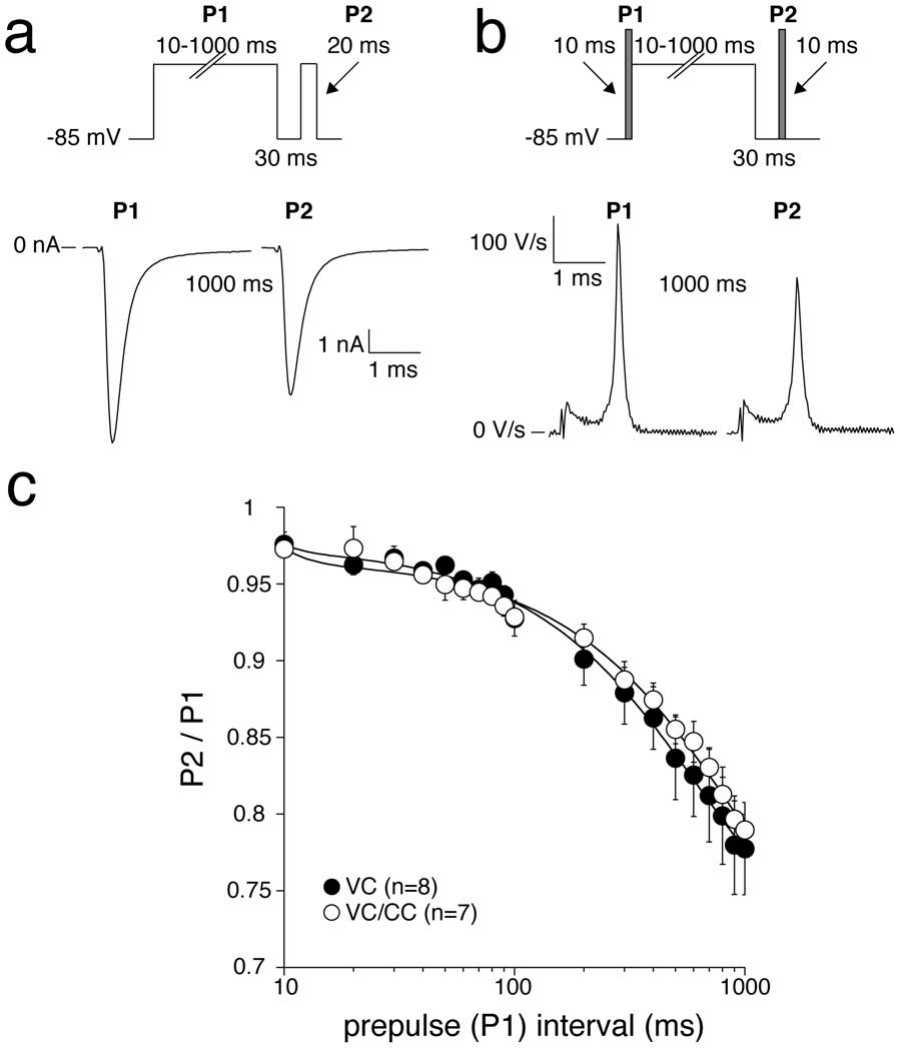
Slow inactivation properties in HEK cells assessed with VC (A) and alternating VC/CC (B). **a and b**, Top: voltage clamp (A) and VC/CC (B) protocols, with a conditioning prepulse interval (P1) of 10–1000 ms, as indicated, and a 30-ms interval to remove fast inactivation. Bottom: typical examples of slow inactivation with a conditioning prepulse of 1000 ms. **c**, Average development of slow inactivation. Note the logarithmic time scale. Solid lines are double-exponential fits to the average data.

Slow inactivation was measured using a two-pulse protocol, where a conditioning prepulse to −20 mV (P1) of variable duration ranging between 10 and 1000 ms was followed by a test pulse (P2) after a 30-ms step to −85 mV to allow the channels to recover from fast inactivation (Fig. 5, a and b, top). Figures 5a and 5b, bottom, show typical P1 and P2 currents and dV/dt values, respectively, with a P1 duration of 1000 ms. The peak amplitudes in response to P2 were normalized to the peak amplitudes at P1, plotted versus the duration of P1, and designated peak I_Na_ (P2/P1). Consequently, the fraction that entered slow inactivation equals 1 – P2/P1. The amount of Na^+^ channels that entered slow inactivation was similar in VC and VC/CC experiments (Fig. 5c and Table 1).

### Properties of I_Na_ at close-to-physiological conditions in cardiac myocytes

In the experiments above, we found that the biophysical properties of I_Na_ in HEK cells under close-to-physiological conditions were nearly identical in VC and alternating VC/CC experiments. These results demonstrate that the alternating VC/CC technique is a suitable method for determining I_Na_ properties under close-to-physiological conditions. To demonstrate the applicability of the technique with freshly isolated cardiac myocytes, we used the VC/CC approach to characterize I_Na_ in rabbit left ventricular myocytes isolated from healthy animals (Ctrl) and from those with heart failure (HF).

**Figure 6:**
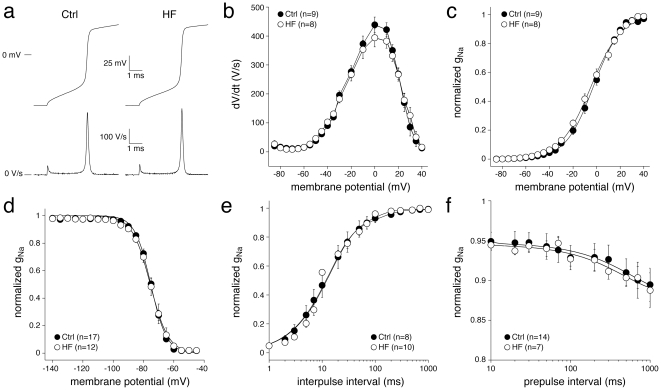
I_Na_ properties in control (CTRL) and heart failure (HF) rabbit ventricular myocytes assessed with alternating VC/CC, using the protocols shown in Figs. 1–5. **a**, Typical examples of AP upstrokes (top) and their dV/dt's (bottom). **b**, Current-voltage relationships of the AP upstrokes. **c**, Dynamic I_Na_ activation curve with Boltzmann fits in solid lines. **d**, Steady-state voltage-dependence of inactivation with Boltzmann fits in solid lines. **e**, Recovery from inactivation with double-exponential fits in solid lines. **f**, Development of slow inactivation with double-exponential fits in solid lines.

Figure 6 shows typical AP upstrokes (panel a), their phase plane plots (panel b), dynamic activation (panel c), inactivation (panel d), recovery from inactivation (panel e) and slow inactivation characteristics (panel f) in Ctrl and HF myocytes, whereas Table 1 summarizes the average biophysical parameters of I_Na_ in Ctrl and HF myocytes. The dV/dt amplitudes did not differ significantly between Ctrl and HF (Fig. 6b) and the voltage dependence of dynamic activation was similar in both groups (Fig. 6c). Moreover, voltage dependence of inactivation as well as recovery from inactivation was similar in Ctrl and HF myocytes. Finally, slow inactivation did not differ significantly between Ctrl and HF. Despite the clearly developed heart failure of our animal model, none of the biophysical properties of I_Na_ differed between Ctrl and HF myocytes under close-to-physiological conditions.

Due to the close-to-physiological temperature and ion concentrations, and to the absence of specific ion channel blockers, we cannot exclude interference of other ion currents, specifically the transient outward K^+^ current (I_to1_) and the L-type Ca^2+^ current (I_Ca,L_), during the dV/dt_max_ measurements in cardiac myocytes of Fig. 6. Therefore, we studied the effects of the I_Ca,L_ blocker nifedipine (500 nmol/L) and the I_to1_ blocker 4-aminopyridine (0.5 mmol/L) on dV/dt_max_. Neither nifedipine (399±10 (nifedipine) vs. 395±7 V/s (control), n = 5) nor 4-aminopyridine (399±31 (4-aminopyridine) vs. 401±27 V/s (control), n = 6) affected dV/dt_max_.

## Discussion

In cardiac myocytes, reliable VC measurements of I_Na_ at close-to-physiological conditions are virtually impossible due to the large amplitude and rapidly activating nature of I_Na_, which seriously hamper the quality of voltage control over the membrane. With the increasing need for accurate measurements of the biophysical properties of I_Na_ under close-to-physiological conditions in cardiac myocytes, we therefore sought for a relatively simple technique that would allow the acquisition of such data, which we found in the alternating VC/CC technique. In this technique, there are no problems with voltage control, because dV/dt_max_, which is proportional to the Na^+^ ion current, is acquired during the CC period. Yet, cells are clamped with protocols similar to those used in conventional VC experiments, allowing acquisition of biophysical parameters as in conventional VC.

In this paper we describe the use of alternating VC/CC experiments for investigating biophysical properties of I_Na_ under close-to-physiological conditions in detail. In HEK cells heterologously expressing SCN5A current, the conventional VC and the alternating VC/CC experiments resulted in similar SCN5A current characteristics, thus validating the alternating VC/CC technique as an alternative to conventional VC for determining biophysical properties of I_Na_. Moreover, we demonstrate that the alternating VC/CC technique is a suitable method for determining I_Na_ properties under close-to-physiological conditions in cardiac myocytes.

### dV/dt_max_ as a tool in studying Na^+^ channel properties

In the past, dV/dt_max_ during an action potential upstroke was used to determine: i) I_Na_ density [48]; ii) I_Na_ activation properties (resulting from plotting dV/dt versus the membrane potential in phase-plane plots [46]); iii) I_Na_ inactivation properties, also called membrane “responsiveness” [31], [49]–[51]; iv) recovery from I_Na_ inactivation properties [52], [53]; and v) development of slow inactivation of I_Na_ [33]. Even though the use of dV/dt_max_ as an index of I_Na_ provided us with important information on Na^+^ channel behaviour, dV/dt_max_ has often been regarded as a nonlinear representation of the underlying Na^+^ channel activity [34], [37], [54]. This argument and the refinement of the VC technique, has shifted attention away from the use of dV/dt_max_ as an index of I_Na_. However, with the need for reliable I_Na_ measurements at both physiological temperature and physiological Na^+^ gradients in cardiac myocytes, the method was recently reintroduced [55], [56].

Previous VC experiments performed in HEK cells demonstrated that heterologously expressed SCN5A currents can reliably be characterized under close-to-physiological conditions [11]–[17]. In the present study we show that SCN5A current properties determined from the analysis of dV/dt_max_ (VC/CC) and those determined in conventional VC closely match (Figures 1, 2, 3, 4, 5, Table 1). This finding is in line with results of Hondeghem and coworkers [25], [33], [35], [57], but contrasts findings of Tsien, Sheets, and their coworkers [18], [20], [37], [54], which support the nonlinear relationship between dV/dt_max_ and the underlying I_Na_. It should, however, be noted that the nonlinearity between I_Na_ and dV/dt_max_ becomes smaller at higher temperatures [37]. While the studies of Tsien and coworkers were - for methodological reasons - executed at low temperatures [18], [20], [37], [54], we were able to perform our experiments at 36°C. This may explain why we did not observe indications for a nonlinear relationship, if any, between dV/dt_max_ and the underlying I_Na_.

Although SCN5A current properties determined from the analysis of dV/dt_max_ (VC/CC) and those determined in conventional VC closely match (Figures 1, 2, 3, 4, 5, Table 1), we found a tendency of smaller peak Na^+^ current in VC/CC compared to VC experiments (cf. Table 1). Although the differences did not reach statistical significance, the smaller peak current may be due to some channels going into an inactivated state during the CC period of the VC/CC technique. Furthermore, we found a significantly steeper dynamic I_Na_ activation curve in our VC/CC experiments (cf. Fig. 2d, Table 1). This might be due to passive membrane loading in the CC period of the VC/CC technique, which suppresses the early phase of dynamic activation. This process does not affect the dV/dt_max_ and therefore does not influence the analyzed biophysical properties.

### Na^+^ current properties in Ctrl and HF myocytes under close-to-physiological conditions

The reported effects of HF on cardiac Na**^+^** current characteristics are not consistent (see Ref. [29], and primary refs cited therein). The discrepancies between the various studies may be due to differences in the methods used to induce HF, in species, or in tissues studied [29]. In the present study, we compared I_Na_ properties in Ctrl and HF myocytes using the alternating VC/CC technique to demonstrate its usefulness for I_Na_ characterization under close-to-physiological conditions in cardiac myocytes. Below, we compare our findings with those from other studies with rabbit HF models.

Using a well-characterized rabbit model of pressure and volume overload-induced HF, we found that all analyzed biophysical properties of I_Na_ were similar in Ctrl and HF myocytes. This agrees with results of conventional VC obtained at room temperature in the same model of HF [55], [58], [59]. Although this pressure and volume overload rabbit model HF is not associated with a ‘persistent’, ‘late’ or ‘sustained’ I_Na_ [58], the VC/CC technique is not suitable for characterization of sustained I_Na_. However, the relatively slow and small sustained I_Na_ can be measured accurately in cardiac myocytes as tetrodotoxin-sensitive current in conventional VC under close-to-physiological conditions [26].

Using HEK cells as an expression system that allows a direct comparison to data from conventional VC, we have shown that biophysical properties of I_Na_, including current density, voltage dependent (in)activation, development of inactivation, and recovery from inactivation, can be accurately determined through alternating VC/CC experiments under close-to-physiological conditions. Thus, our study demonstrates that the alternating VC/CC technique is a valuable experimental tool for I_Na_ measurements in cardiac myocytes under close-to-physiological conditions.

### Technical considerations

In the present study, we used a custom-made patch clamp amplifier to perform the alternating VC/CC technique. Nowadays, sophisticated commercially available patch clamp amplifiers also exhibit the possibility to make a computer-controlled rapid switch between recording modes (VC or CC), thus allowing a straightforward implementation of the VC/CC technique. These include widely used amplifiers from major manufacturers, such as the MultiClamp 700B from Molecular Devices (Sunnyvale, CA, USA), the RK-400 from Bio-Logic (Claix, France), the VE-2 Whole-Cell Patch Clamp Amplifier from Alembic Instruments (Montreal, Quebec, Canada), and the EPC 10 USB Patch Clamp Amplifier family from HEKA (Lambrecht/Pfalz, Germany).

### Limitations

While the alternating VC/CC technique is an appropriate method to characterize I_Na_ density and gating properties, it does not allow the estimation of late or persistent I_Na_. However, as set out above, this sustained I_Na_ can be measured accurately in cardiac myocytes as tetrodotoxin-sensitive current in conventional VC under close-to-physiological conditions. Furthermore, due to the close-to-physiological temperature and ion concentrations, and to the absence of specific ion channel blockers, we cannot exclude interference of other ion currents, specifically the transient outward K^+^ current (I_to1_) and the L-type Ca^2+^ current (I_Ca,L_), during the dV/dt_max_ measurements in cardiac myocytes. However, it may be argued that the contribution of I_to1_ and I_Ca,L_ to the cardiac action potential upstroke is limited for several reasons. First, current through Na^+^ channels in myocytes has a relatively high density compared to other channels; in our hands, average dV/dt_max_ in rabbit ventricular myocytes was around 400 V/s (Fig. 6b), which corresponds to a 400 pA/pF current. Second, action potential upstrokes in ventricular myocytes are extremely fast, with an upstroke duration of approximately 0.5 ms (Fig. 6a), a time window during which I_to1_ and I_Ca,L_ exhibit negligible activation. In line with these arguments, we found no significant effects of the I_to1_ blocker 4-aminopyridine and the I_Ca,L_ blocker nifedipine on the dV/dt_max_ in cardiac myocytes.

### Conclusions

The behaviour of Na^+^ channels in cell expression systems differs from that in cardiac myocytes. Therefore, the need for accurate I_Na_ measurements under close-to-physiological conditions in myocytes of healthy animals and in myocytes of animal models with various cardiac disorders is increasing. Changes in gating properties of Na^+^ channels due to mutations may be more prominent at near-physiological temperature than at room temperature. Our study demonstrates that the alternating VC/CC technique facilitates successful analysis of Na^+^ channel properties in cardiac cells under close-to-physiological conditions. In addition, the analysis of dV/dt immediately unravels the physiological consequences of altered Na^+^ channel properties. Overall, due to its simplicity and convenience, the alternating VC/CC technique is a valuable experimental tool in cardiac cellular electrophysiology.
